# Investigation of EBT3 radiochromic film's response to humidity

**DOI:** 10.1002/acm2.12337

**Published:** 2018-04-29

**Authors:** Elsa Y. León‐Marroquín, José M. Lárraga‐Gutiérrez, J. Alfredo Herrera‐González, Miguel A. Camacho‐López, José E. Villarreal Barajas, Olivia A. García‐Garduño

**Affiliations:** ^1^ Laboratorio de Fotomedicina, Biofotónica y Espectroscopia Láser de Pulsos Ultracortos, Facultad de Medicina Universidad Autónoma del Estado de México Toluca México; ^2^ Laboratorio de Física Médica & Unidad de Radiocirugía Instituto Nacional de Neurología y Neurocirugía Mexico City México; ^3^ Departamento de Oncología & Departamento de Física y Astronomía Universidad de Calgary Calgary AB Canada

**Keywords:** EBT3 radiochromic film, humidity, net optical density

## Abstract

**Purpose:**

The aim of this work is to investigate the effects of immersing EBT3 radiochromic film in water and to evaluate its contribution to the total uncertainty in dose determination.

**Materials and methods:**

We used 3 cm × 3 cm EBT3 radiochromic films irradiated in the range of 0–70 Gy to study the impact of water immersion on the change in net optical density. These films were placed in a water container for a period of 24 h. The net optical density was measured before (0 h) and after of the immersion in water (1, 3, 6, 12, 18, and 24 h). The absorbance spectrum of the EBT3 radiochromic film was measured at 0 h and 24 h after immersion in water. The uncertainty in dose determination due to the effects of keeping the EBT3 radiochromic film submerged in water at 0, 1, and 24 h were recorded in the red, green, and blue channels.

**Results:**

We observed an increase in the net optical density as an effect on the film due to its immersion in water. The penetration of the water at the edges of the radiochromic film was observed to be a function of time during which the film remained in the water. On the other hand, the penetration of water at the edges of the film was found to be independent of irradiation dose.

**Conclusions:**

EBT3 radiochromic film is found more resistant to water penetration through the edges than its predecessors. However, there is evidence that suggest that liquid water damage the Nylon cover layer of the film by changing its optical properties. Therefore, it is recommended to build a new calibration curve for radiochromic films for a specific situation involving dose measurements in liquid water.

## 1 INTRODUCTION

Radiochromic film is a two‐dimensional (2D) radiation detector with high spatial resolution used in radiotherapy, primarily for research and quality assurance, or quality control measurements.^1^ According to the manufacturer, the EBT3 radiochromic film is an improved version of the EBT2 radiochromic film. It is made by laminating an active layer between two identical polyester layers, which makes the film more robust and allows water immersion.^2^ In addition, its symmetrical structure allows for analysis independent of the film side orientation, a feature which is not present in the case of the EBT2 radiochromic film. Another advantage of the EBT3 radiochromic film is that it is less sensitive to indoor lighting. Previous studies have investigated characteristics of the EBT3 radiochromic film including its the energy dependence for x‐ray irradiation,^3^, ^4^, ^5^ particle‐type dependence,^6^ its utility in intensity‐modulated radiotherapy (IMRT) applications,^4^ its compared with its predecessor (the EBT2 radiochromic film)^7^, ^8^ and spectral analysis of EBT3 film for 6‐MV photon beams.^9^

Radiochromic films offer a key advantage in that they can be submerged in water. Even though it is true that the current use of radiochromic film may not require a long‐time immersion of film pieces in water, reference dosimetry for high‐dose and low‐dose rate brachytherapy sources in water may require submerging the film pieces in water for hours.^10^

There are studies that have reported the impact of radiochromic film immersion in water. In 2001, Butson et al.^11^ investigated the effects of water medium on the MD‐55‐2 radiochromic film. Their results showed a small penetration rate of water into the film, which only affected the outer areas of the film, with penetration being <0.5 mm/h. They also reported that the optical density of the film at the center remained unchanged after it was removed from water. These results suggest that the edge of the film where visible penetration has occurred should be discarded. However, other areas with the film are not affected when the film is placed in a water phantom. They concluded that the radiochromic film was an adequate detector for dosimetry in a water phantom where high spatial resolution is necessary and the angle of beam incidence at the point of interest is important.

Van Battum et al.^1^ studied the dosimetric properties of Gafchromic EBT film and compared it with the ionization chamber data in water. Their results showed that after 15 min of immersion in water, a slight light blue (approximately 2 mm thick) fog along the film edges was visible due to water penetration. After drying, with the film kept at room temperature for 1 h, no such water trace was detectible even after scanning.

Rink et al.^12^ investigated the dependence of the response of the EBT film on temperature and its state of hydration. They found that the sensitivity of the EBT film model to ionizing radiation is also a function of the hydration of the sensitive layer. They concluded that the water influences the three‐dimensional structure of the monomer crystals and desiccating the samples shifted both the absorbance peak to a higher wavelength and decreased sensitivity.

Aldelaijan et al.^10^ investigated the impact of the EBT2 radiochromic film's immersion in water for various parameters: the impact of the film piece size, initial optical density, postimmersion waiting time prior to scanning, and the time during which the film was kept in water. Moreover, they investigated the pathways of water penetration into the film during the film immersion in water. They found that the penetration depth could reach up to 9 mm around the edges of the EBT2 radiochromic film. The anticipated dose error due to the change in optical density because of the water immersion appears to be negligible for the short immersions of the order of 30 min. However, as the immersion time increases, the anticipated dose error may reach 7% at 3 Gy of measured dose. Moreover, their results showed that the net absorption change due to the water only is more dominant around the main absorption peaks, centered around 583 and 634 nm. This result suggests that there might be an optical density change, which must be accounted for if accurate dose measurements are to be performed with pieces of the EBT2 model radiochromic film. However, this does not appear to be necessary for the case of the blue channel (400–500 nm), in which the change is shown to be uniform and independent of dose. They also found that the water does not only diffuse through the edges of the film piece, but also through the protective polyester sides of the film at the very same rate as the diffusion through the film edges. The authors suggested various approaches in correcting for the changes in net optical density (netOD) due to water penetration into the film, which must be incorporated into the current film dosimetry protocol. However, they believe that the use of the control film piece would be the most appropriate method.

Recent studies using Monte Carlo simulation have proven that the EBT3 film model has energy dependence with the influence of the phantom material on the absorbed dose. Chan et al. proved the efficiency of EBT3 film for a commercial electron Monte Carlo dose calculation algorithm. The work highlights the potential for significantly changing the actual dose delivered to patients if dose to medium is calculated rather than dose to water.^13^ Hermida et al. found this model of radiochromic film EBT3 the maximum difference of energy dependence for the solid phantom respect to water was about 6% at energy of 50 keV and attribute to overall energy dependence of the EBT3 film in water is mainly due to its intrinsic energy dependence.^14^

Considering the above, especially the change in netOD that can present some models of radiochromic films, the aim of this work is to investigate the effects of the EBT3 radiochromic film's immersion in water. This work analyzes the changes in netOD, penetration, and penetration rate. In addition, the net absorption spectra is analyzed. Furthermore, by propagation of error, an uncertainty analysis is performed to evaluate the contribution to the total uncertainty in determining the dose.

## 2 MATERIALS AND METHODS

### 2.A Film irradiation

Gafchromic^®^ EBT3 (Gafchromic, Ashland Specialty Ingredients, NJ, USA) films with Lot# A01171301 were used to study the impact of water immersion on the net optical density. The films were handled according to the procedures described in the AAPM Task Group # 55 report.^15^ Radiochromic films of 3 cm × 3 cm size were placed in the center of a solid water phantom (CIRS Inc., Norfolk, VA, USA) at 5 cm depth and were perpendicularly irradiated with a 6‐MV photon beam using a Novalis^®^ LINAC linear accelerator (Novalis, BrainLab, Germany). The LINAC was calibrated such that a 1 cGy per monitor unit is delivered at a 10 cm × 10 cm field size and a source‐to‐surface distance of 95 cm. Four radiochromic films were irradiated for each dose between 0 and 70 Gy in order to reduce the statistical uncertainty,^16^ considering the following doses: 0, 1, 3, 6, 15, 35, 50, and 70 Gy.

### 2.B Film immersion in water

After irradiation, radiochromic films were stored 72 h until they reach color stabilization according with TG‐55.^15^ The effects of water immersion time on the radiochromic film response were studied by placing the films in a container filled with water for a period of 24 h. During this period, the radiochromic film was scanned at intervals of 1, 3, 6, 12, 18, and 24 h. This was performed by taking the film out of the water and towel‐drying it before readout.

### 2.C Scanning procedure and analysis

**Figure 1:**
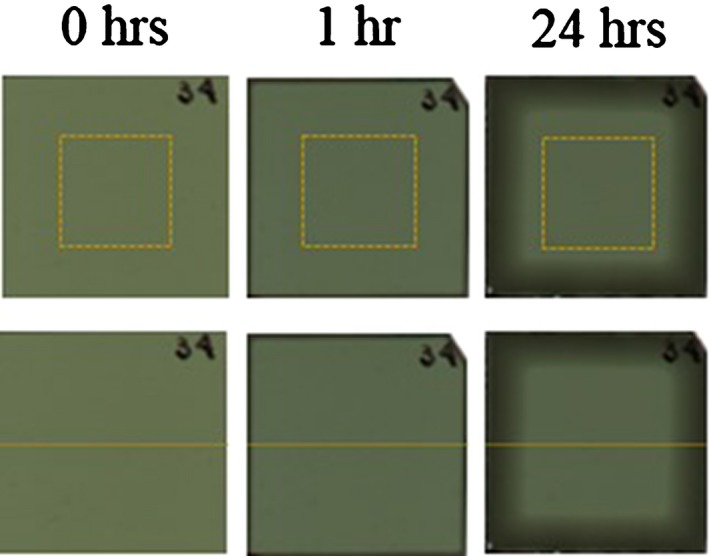
(a) Region of interest (ROI) of 1.5 cm × 1.5 cm that we considered to obtain the values of the transmitted light intensity (*I*) and standard deviation associated with this value (SD *(I)*). (b) Measure of profiles of the radiochromic film.

An Epson Perfection V750 desktop scanner was used for scanning the EBT3 radiochromic films. Radiochromic films were scanned using an Epson scan software in RGB‐positive mode at a depth of 16 bits per color channel and a spatial resolution of 72 dpi (that corresponded to a pixel size of 0.35 mm × 0.35 mm) without applying any image processing features. The radiochromic films were positioned in the center of the scanner bed and in portrait mode. The images were saved in TIFF format. Subsequently, the images were imported for processing using commercial software ImageJ (v.1.2). This work considered a region of interest of 1.5 cm × 1.5 cm in the center of the radiochromic film, avoiding the zone of penetration of the water as shown in Fig. 1(a), to obtain the values of the transmitted light intensity (*I*) and the standard deviation associated with this value (SD*(I)*).

In this work, it was used three definitions of net optical density (netOD). The first definition is the following:

(1) $$\begin{array}{c} {\text{netOD}}_{d,d}=-{\text{log}}_{10}\frac{I_{\mathrm{dry}}}{I_{0,\mathrm{dry}}} \end{array}$$

where *I*_dry,_ and *I*_*0,*dry_ are pixel intensity values for irradiated and nonirradiated films, respectively. The Eq. (1) assessed the film response in dry environment. The second definition is:

(2) $$\begin{array}{c} {\text{netOD}}_{w,d}={\text{log}}_{10}\frac{I_{\mathrm{wet}}}{I_{0,\mathrm{dry}}} \end{array}$$

where *I*_wet_ and *I*_0,dry_ dry are pixel intensity values for irradiated in a wet and nonirradiated films in a dry environment, respectively. The Eq. (2) assessed the effect of water immersion on the film response relative to a dry background film for a fixed immersion time. The last definition is:

(3) $$\begin{array}{c} {\text{netOD}}_{w,w}=-{\text{log}}_{10}\frac{I_{\mathrm{wet}}}{I_{0,\mathrm{wet}}} \end{array}$$

where *I*_wet_ and *I*_0,wet_ are the pixel intensity values for irradiated and nonirradiated films in a wet environment, respectively. The Eq. (3) assessed the changes of the sensitometric curve for a fixed immersion time.

Their associated standard deviation is the following general expression:

(4) $$\begin{array}{c} \text{SD}\;\left(\text{netOD}\right)=\frac{1}{\text{ln}10}\sqrt{{\left(\text{SD}\left(I_{x}\right)/I_{x}\right)}^{2}+{\left(\text{SD}\left(I_{o,y}\right)/I_{o,y}\right)}^{2}} \end{array}$$

where the indexes *x* and *y* stand for wet and dry films when applicable for Eqs. (1), (2), (3). A set of sensitometric curves were assessed as a function of water immersion time for dry, 1 and 24 h for comparison.

Also, it was study the evolution of optical density (OD) in the central region as a function of immersion time. Moreover, in this case it was defined OD as follows:

(5) $$\text{OD}=-{\text{log}}_{10}\left(\frac{1}{2^{16}}\right)$$

where 2^16^ is the maximum transmittance of the scanner and *I* is the film reading for a particular dose and immersion time. In this case, the associated uncertainty to Eq. (5) was,

(6) $$\text{SD(netOD)}=\frac{1}{\text{ln}10}\frac{\text{SD}(I)}{I}$$

### 2.D Profiles

Film response profiles were measured as shown in Fig. 1(b). We found that the damage caused by the cut of the EBT3 film was to be 2 mm on each side. Therefore, the profiles were measured for a length of 2.6 cm. The intensity profiles were converted to netOD_w,d_ by using Eq. (2). The profiles were averaged to measure the penetration of water from film edge and to assess the penetration velocity as the ratio between penetration distance and immersion time.

### 2.E Absorbance spectrum

To measure the change in absorbance spectrum of the EBT3 radiochromic film, the absorption spectra were measured before (0 h) and 24 h after immersion in water using a PerkinElmer UV/Vis Lambda 650 double‐beam spectrophotometer (Perkin Elmer, Waltham, MA, USA). The radiochromic films are typically positioned perpendicular to the direction of the light beam. The absorption spectrum of each irradiated EBT3 radiochromic film at 1, 6, and 35 Gy was taken by placing on the right holder in the center of the radiochromic film.

### 2.F Uncertainty analysis

To evaluate the uncertainty in the determination of the dose due to the effects of keeping the EBT3 radiochromic film submerged in water at 0, 1, and 24 h in the three color channels: red, green, and blue, we follow the methodology proposed by Devic et al.^17^ To obtain the dose–response, an analytical expression was obtained by fitting the data using the least squares method for each color channel:

(7) $$D=a\times \text{netOD}+b\times {\text{netOD}}^{n}$$

Next, the uncertainty in determining the dose was calculated using an error propagation analysis.^17^ The total scan uncertainty (*SD*_*tot*_) was calculated using the following expression:

(8) $${\text{SD}}_{\mathrm{tot}}=\sqrt{{\text{SD}}_{\exp}^{2}+{\text{SD}}_{\mathrm{fit}}^{2}}$$

where SD_exp_ is the experimental uncertainty and SD_fit_ represents the fitting uncertainty.

## 3 RESULTS AND DISCUSSION

### 3.A Profiles

**Figure 2:**
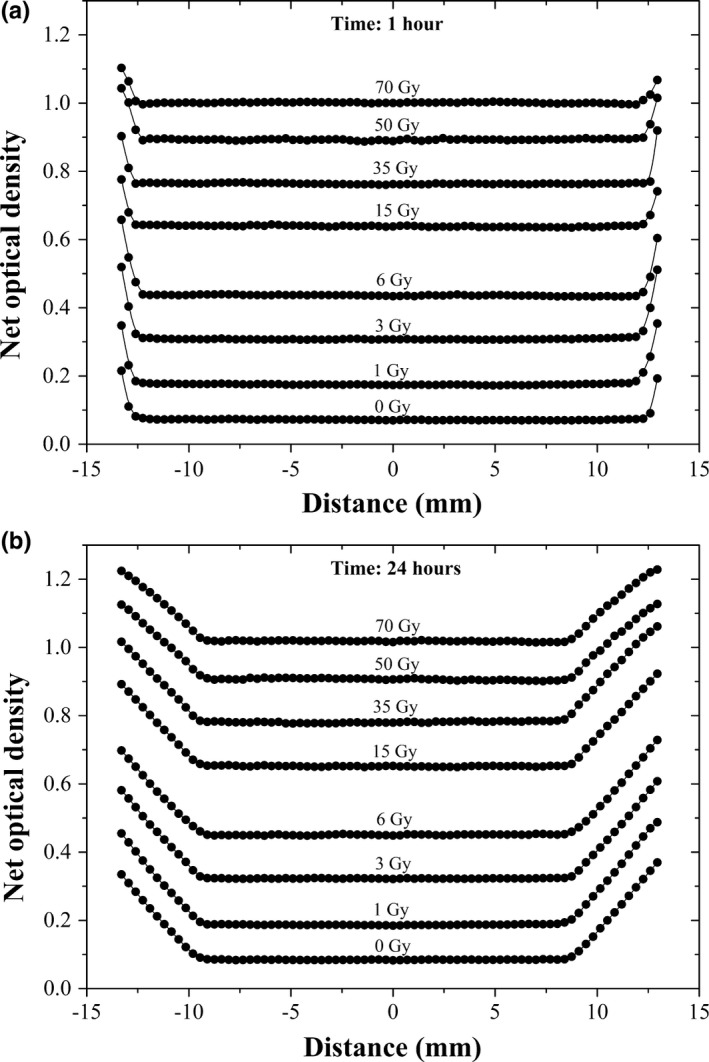
Results of net optical density profiles (netOD_w,d_) of the EBT3 radiochromic films irradiated from 0 to 70 Gy after being submerged in water for 1–24 h.

**Figure 3:**
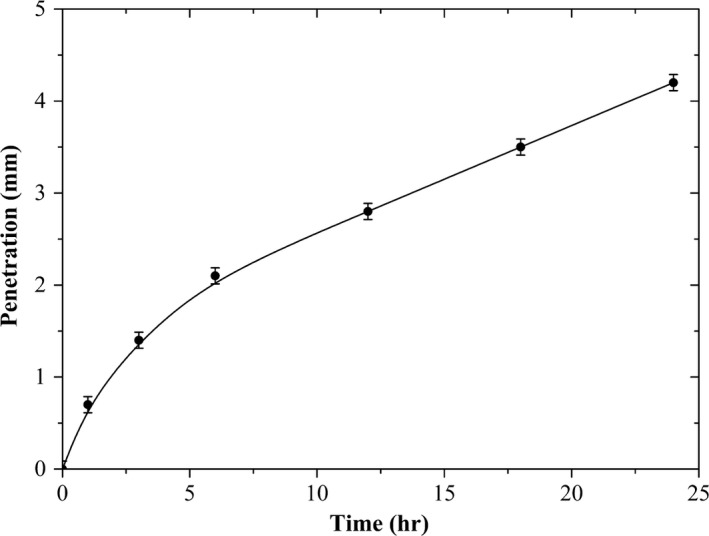
Penetration of water into the edges of the EBT3 radiochromic film.

**Figure 4:**
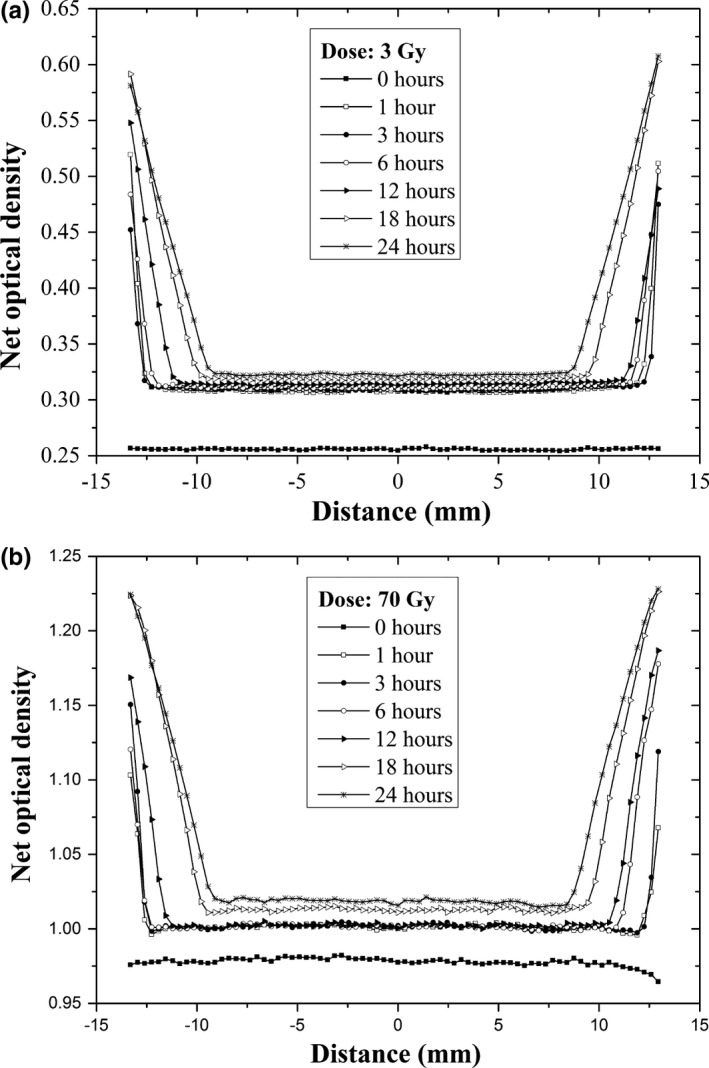
Results of the net optical density profiles (netOD_w,d_) of the EBT3 radiochromic films immersed in water during 0–24 h to irradiation dose of 3–70 Gy.

Figures 2, 3, 4 show the effects of immersion time in the water onto to the EBT3 radiochromic film response (net optical density) irradiated at different dose levels between 0 and 70 Gy for the red component. Figure 2 shows the results of the net optical density profiles of the films irradiated for 0–70 Gy for an immersion time of 1–24 h. An increase in the penetration of the water at the edges of the radiochromic film can be clearly seen as a function of time during which the film remained in the water. This penetration goes from 0.70 to 4.20 mm after remaining in water for 1–24 h. Nevertheless, the penetration of water at the edges of the film is independent of the dose of irradiation.

Figure 3 shows the penetration of the water into the edges of the EBT3 radiochromic film as a function of time. It can be observed the water penetrates at a decreasing rate with time. After 24 h, during which the EBT3 radiochromic film remained submerged in the water, we found that the penetration was 4.20 ± 0.09 mm. These results were consistent with similar results reported in the literature.^10^, ^11^

However, the velocity decreases with time when the radiochromic film remained in the water. This is because the polymer density is greater as the distance through the water flows. It would even seem that the velocity of penetration tends to be constant with the time of submersion as shown in Table 1.

**Table 1:** Penetration rate of water at the edges of EBT3 radiochromic film. The standard deviation is showed between parenthesis. The uncertainty associated to time measurements was fixed <2%. The results were rounded

*T* (h)	*D* (mm)	*V* (mm/h)
0	0	0
1	0.70 (24%)	0.70 (24%)
3	1.40 (27%)	0.47 (27%)
6	2.10 (19%)	0.35 (19%)
12	2.80 (16%)	0.23 (16%)
18	3.50 (12%)	0.19 (12%)
24	4.20 (9%)	0.18 (9%)

Figure 4 shows the effects of water on the radiochromic film as an increase in the net optical density between the radiochromic films without immersing them in water (0 Gy), and those that are immersed in water for a certain period of time (1, 3, 6, 12, 18, and 24 h). We found that an increase in the net optical density is 16.8% for 3 Gy and 2.3% for 70 Gy, between the radiochromic films not immersed in water (0 h) and those that were immersed in water for 1 h. The net optical density was measured in the center of the film where the water did not penetrate. The increase in the net optical density for the films remaining in the water for 1 and 24 h is 4.8 and 1.6% for 3 and 70 Gy, respectively.

Table 2 shows the change in the net optical density for the radiochromic films belonging to the red component before submerging it in water and also after remaining in the water for 1 h (ΔnetDO_1–0 h_). This also shows the changes in the films that were submerged in the water for 1–24 h (ΔnetDO_24–1 h_). It is observed that, ΔnetDO_1–0 h_ is greater than ΔnetDO_24–1 h_. In addition, we can observe that ΔnetDO_1–0 h_ decreases as a function of the dose, whereas ΔnetDO_24–1 h_ tends to be constant with the dose. The net optical density was measured in the center of the film where it was prevented from water penetration.

**Table 2:** Changes in the net optical density of the radiochromic film for the red component: difference between before submerging it in water and after remaining in the water for 1 h (ΔnetDO_1–0 h_), and changes between the films that remain submerged in the water for 1–24 h (ΔnetDO_24–1 h_)

Dose (Gy)	ΔnetDO_1–0 h_	ΔnetDO_24–1 h_
0	0.071	0.013
1	0.064	0.013
3	0.052	0.015
6	0.044	0.014
15	0.033	0.013
35	0.029	0.014
50	0.024	0.015
70	0.023	0.017

**Figure 5:**
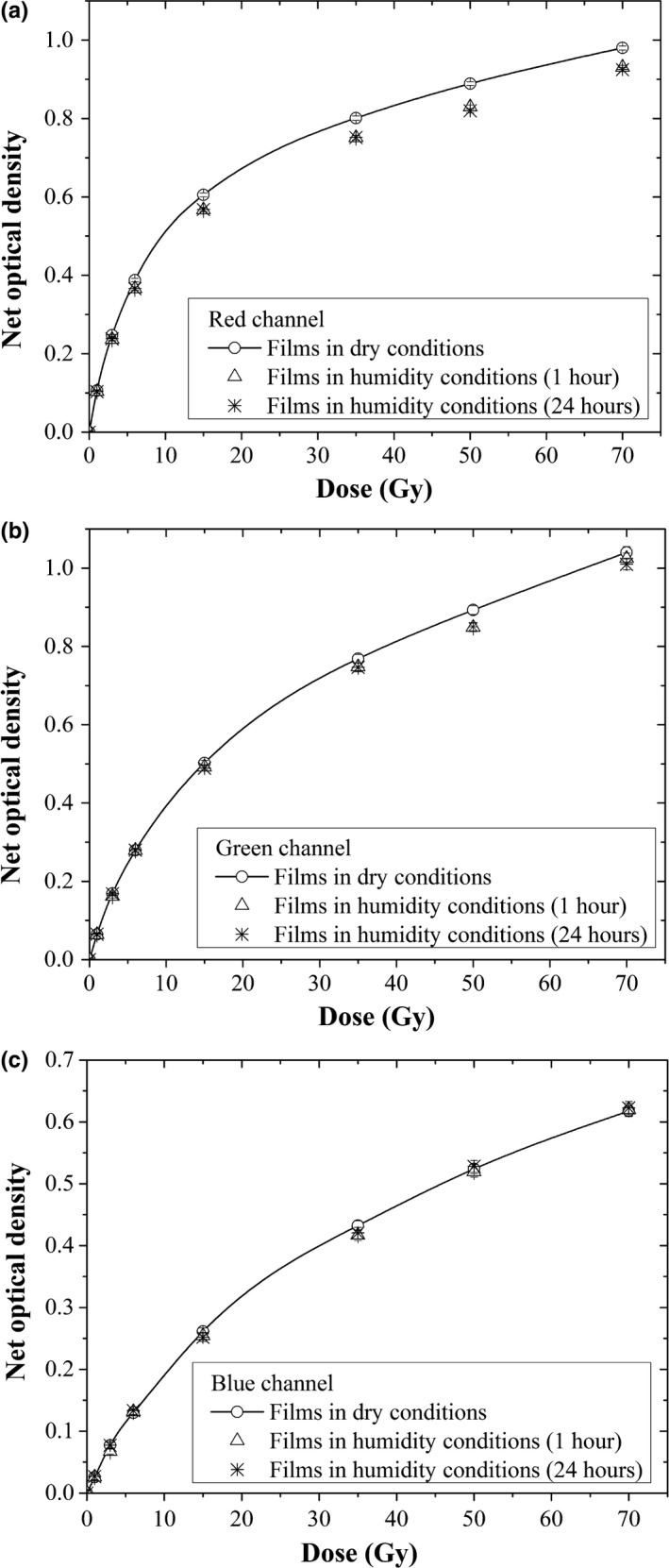
Sensitometric curves of the EBT3 radiochromic films before being immersed in water (netOD_d,d_) and after remaining in the water for 1–24 h (netOD_w,w_), for three color channels: a. red, b. green, and c. blue.

Figure 5 shows the sensitometric curves of EBT3 radiochromic films before being immersed in water (dry) and after remaining in the water for 1 and 24 h for each color channel. The net optical density was measured in the center of the film that was prevented from water penetration. It can be observed that the net optical density of the film increases when it is immersed in water. In addition, it can be seen that the change in optical density (ΔnetOD) for the red channel (Fig. 5a), between the film before being immersed in water and after remaining in the water for 1 h, is greater than for the green (Fig. 5b) and blue (Fig. 5c) channels. The optical density net changes are, approximately, of 3.1%, 1.6%, and 0.6% for the red, green, and blue channels, respectively. We also observed that the change in the net optical density between the films remaining in the water for 1–24 h is small, resulting from 0.2% for the red channel 0.3% for the green channel; while for the blue channel it is 0.6%.

**Figure 6:**
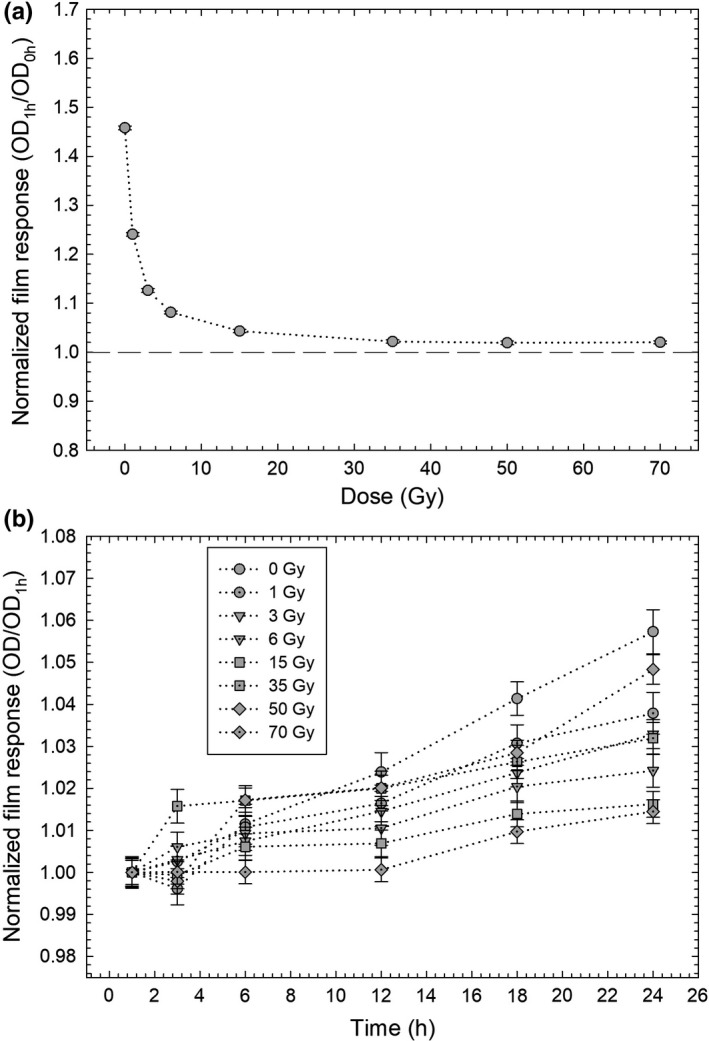
Evolution of film's OD as a function of immersion time. (a) OD normalized relative to the response of a 0 h film and (b) OD normalized relative to the first hour of immersion time. Dotted lines are only for visual guidance.

Figure 6 shows the evolution of OD as a function of immersion time for the central region of the film. In Figs. 6(a) and 6(b), film's OD was normalized relative to 0 h immersion time (Fig. 6a) and relative to 1 h immersion time (Fig. 6b). It can be observed in Fig. 6(a) that there is great change in film's OD (between 10% to 50%, approximately) in the first hour of water immersion. This change is more appreciable for films irradiated with doses <5 Gy. For doses >5 Gy, the film's OD changes less or equal than 2%. For water immersion times >1 h (Fig. 6b), there are changes in film's OD <6% for a 24‐h immersion time.

### 3.B Absorbance spectrum

**Figure 7:**
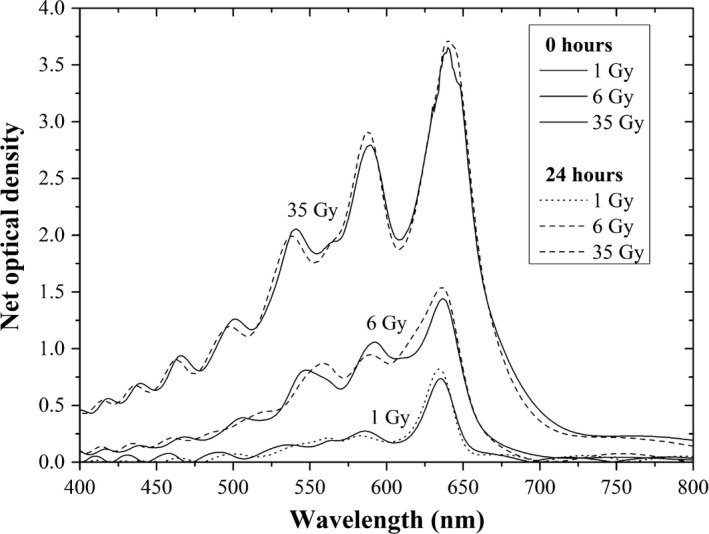
Net absorption spectra of the EBT3 radiochromic films before (0 h) and after 24 h immersion in water.

Figure 7 show the net absorption spectra of the EBT3 radiochromic films before being immersed in water (0 h) and after remaining in the water for 24 h. Observing the absorption band centered around 636 nm, we note that the net optical density of the radiochromic film irradiated at 1, 6, and 35 Gy before being immersed in water (0 h) is less than the net optical density of the film after remaining 24 h in water.

### 3.C Uncertainty analysis

Table 3 shows an evaluation of the uncertainty in the determination of the dose for the EBT3 radiochromic film submerged in water at 0, 1, and 24 h and its associated standard deviation for the red component. When the EBT3 radiochromic films (that were submerged in water, for 1–24 h) are decomposed into all three color channels, the observed effect is an increase in the net optical density.

**Table 3:** Evaluation of the uncertainty in the determination of the dose for keeping the EBT3 radiochromic film submerged in water at 0, 1, and 24 h

*T*_Immersion_	0 h		1 h		24 h	
D (Gy)	D_fit_ (Gy)	SD	D_fit_ (Gy)	SD	D_fit_ (Gy)	SD
0	0.00	0.00	0.00	0.00	0.00	0.00
1	0.96	2.63	0.93	3.71	0.93	4.31
3	2.83	2.03	2.65	2.64	2.62	2.86
6	5.99	2.70	5.39	2.81	5.39	2.95
15	14.92	3.22	14.13	4.12	13.97	4.24
35	35.06	4.20	33.96	4.43	33.35	4.67
50	49.89	4.23	49.03	4.50	48.81	4.77
70	70.96	4.20	71.58	4.57	71.73	5.56

## 4 DISCUSSION AND CONCLUSIONS

In this work, we investigated the effects of the EBT3 radiochromic film's immersion in water. Figures 2, 3, 4 show the effects of immersion time on the EBT3 radiochromic film's response (net optical density) irradiated at different dose levels for the red component. An increase in the penetration of water at the edges of the radiochromic film can be clearly identified as a function of time during which the film remained in the water (Figs. 2 and 3). However, the penetration of water at the edges of the film is irradiation dose‐independent. In addition, we observed that water causes an increase in the net optical density of the radiochromic film. The results are consistent with those reported by Butson et al.^11^ where they observed that the water penetration into the edge of the EBT radiochromic film produced an opaque and whitish color. Figure 3 shows the penetration of water into the edges of the EBT3 radiochromic film as a function of time. It is observed that the water penetrates at a decreasing rate with time. These results are consistent with those reported in the previous studies. Butson et al.^11^ reported a penetration of approximately 5 mm for the MD‐55‐2 radiochromic films; while Aldelaijan et al.^10^ found a penetration of 6 mm for the EBT2 radiochromic film (both were for a 24‐h immersion time). Therefore, the results of this work (4.20 ± 0.09 mm) apparently show that the EBT3 radiochromic film is more resistant to water penetration through the edges than its predecessors.

In evaluating the changes in the net optical density of the EBT3 radiochromic film due to water immersion, we found that this effect decreases with the dose for the immersion interval of 0–1 h, while for the immersion interval of 1–24 h it remains almost constant (Fig. 4 and Table 2). It is worth mentioning that the net optical density was measured in the center of the film to avoid the area where the water penetrated. Therefore, the EBT3 radiochromic film does suffer from damage in the texture, contrary to what was observed by Aldelaijan et al.^10^ for the EBT2 radiochromic films. Figure 5 shows the response curves of the EBT3 radiochromic films for three color channels of the flatbed scanner, before being immersed in water and after remaining in water for 1 and 24 h.

It can be observed in Fig. 6 that in the first hour of water immersion there is great change in film's OD (between 10% to 50%, approximately). This change is more appreciable for films irradiated with doses <5 Gy. This behavior may be explained by the following: it is well known that Nylon, which is part of the substrate layers of the radiochromic film, is hydrophilic. The water interacts with the bonds of carbon‐oxygen (C‐O) and nitrogen‐oxygen (N‐H) groups of the Nylon film.^18^ These chemical interactions change the mechanical and it may change the optical properties of the film which are of interest for dosimetry. If we assume that the water did not reach the central portion of the film then the “chemical damage” on the film's Nylon coating occurs in the first hour after water immersion. For 0 Gy film, this “chemical damage” can be observed as an increase in OD which is about 50% of the 0 h film. For later immersion times, the damage exists but compete with the increase in OD due to the ionizing radiation. For higher doses (>15 Gy), the change in OD due to water immersion is about 2% compared to that by the ionizing radiation.

It is observed that the net optical density of the film increases when it is immersed in water. In addition, it can be seen that the change in the optical density for the red channel between the film before being immersed in water and after remaining in the water for 1 h, is greater than that for the green and blue channels. This suggests that the response of the film at the peak of absorption is more sensitive to the effects of water on the film, unlike the blue region of the visible spectrum where the changes are minimal, ~400–540 nm, as corroborated in Fig. 6. This net optical density behavior shows that the response of the EBT3 radiochromic film is dose‐independent as mentioned above.

Finally, the evaluation of uncertainty in dose determination for the EBT3 radiochromic film submerged in water at 0, 1, and 24 h for three color channels showed an increase in dose‐associated uncertainty, which is not desirable for film dosimetry. Therefore, this work suggests that if the radiochromic films are to be used for measurements involving immersing them in water, it is recommended that a calibration curve should be constructed under these conditions to avoid any increase in dose uncertainty.

## CONFLICT OF INTEREST

The authors have no conflicts of interest.
